# Sports injury risk assessment based on a training and functional movement analysis of young elite equestrian athletes– an exploratory cross-sectional study

**DOI:** 10.1186/s13102-025-01138-x

**Published:** 2025-04-14

**Authors:** Alexander Havertz, David Uebis, Rudolph Schifflers, Frank Hildebrand, Christian David Weber

**Affiliations:** 1 Department of Orthopedics, Trauma and Reconstructive Surgery, Medical Faculty, University Hospital RWTH Aachen, Pauwels Strasse 30, 52074 Aachen, Germany; 2 Department of Physical Therapy, University Hospital RWTH Aachen, Pauwels Strasse 30, 52074 Aachen, Germany; 3 CHIO Aachen Medical Center and Olympic Center Rhineland, Aachen, Germany

**Keywords:** Equestrian sports, Functional movement screen, Hip dysbalance, Physical abnormalities, Sports injury, Thomas test, Y-balance test

## Abstract

**Background:**

Dressage and show jumping is a high-risk sport, especially for young and professional riders.

**Objective:**

To analyze hip flexibility and strength, dynamic body balance, functional movement and pelvic obliquity in junior elite equestrian athletes as potential targets for future preventive measures.

**Design:**

A single-center cross-sectional study.

**Methods:**

Members of an elite junior equestrian team (*N* = 12) underwent standardized interviews, basic orthopedic examinations, lower quarter Y-balance testing (YBT-LQ), functional movement screening (FMS), and hip abductor/adductor strength measurements. Analysis of covariance (ANCOVA), a linear mixed model, and univariable logistic regression were used.

**Results:**

General medical issues were reported by 83.3% and orthopedic issues by 66.7% of the participants. For the YBT-LQ test, the mean composite score was 89.6% ± 8.0%, and maximally reached distances in one direction of movement varied between 0.1 and 5.4 cm between the right and left legs. Posteromedial reached distances were significantly influenced (*p* =.031) by years of training in equestrian sports. Participants achieved an average of 15.2 ± 1.9 points in the FMS, and two had scores below 14. Hip strength measurements showed 8-19% stronger adductors than abductors. Hip flexion contractures were identified in all show jumping athletes.

**Conclusion:**

The results focus on the imbalances that can pose a high risk of injury. In particular, in future training concepts and preventive efforts, imbalance should be addressed in the Y-balance test, hip muscles with stronger adductor than abductor, and hip flexion contracture.

**Supplementary Information:**

The online version contains supplementary material available at 10.1186/s13102-025-01138-x.

## Introduction

With 2.32 million active equestrian riders in Germany [1], equestrian sports, such as dressage and show jumping, are popular recreational and competitive activities.

Equestrian riders face the potential danger of overuse injuries and traumatic insults. With an objective injury frequency of between 1 and 2.85 injuries per 1000 h of riding, the injury rate is low [2] (cf. professional soccer, where the injury rate per 1000 h is 8.7 [3]), although the consequences of accidents can be severe.

The most common traumatic injuries suffered by riders are contusions (41.8%), fractures (39%), brain injury (13%) and visceral organ injury (3.1%) [4]. The use of adequate safety clothing in form of helmets resulted in a trend towards fewer head injuries and more extremities injuries considering the relative distribution of traumatic injuries [4, 5]. In comparison, tendinitis of the elbow and carpal tunnel syndrome are the most common types of overuse injuries in show jumping riders (data not available for all riders) [6].

Each rider faces 6.7 ± 17.0 weeks of traumatic injury–related downtime throughout their career [6]. While inexperienced riders have a higher amount of trauma, professional riders have more severe trauma, and riders of older horses have less frequent trauma [5, 6]. Consequently, professional junior riders can be seen as a high-risk group for traumatic events.

In addition to direct trauma mechanisms (e.g., falling off a horse), there are general risk factors for overuse injuries in equestrian sports. Early athletic specialization is a risk factor for these injuries [7, 8]. Equestrian athletes have a special riding position on the horse and must intensely use their adductors to press their legs against the saddle to achieve a stable riding position. This results in the adductors being under more strain than the abductors, which causes muscular imbalance to occur.

Although the literature contains numerous studies of injury foci (e.g., spine and brain injuries) [2, 9, 10], along with research on their mechanisms and the prevention of trauma in equestrian sports, the number of epidemiological studies on equestrian overuse injuries has been described as low [9, 11]. Therefore, more focus should be placed on the prevention of sports overuse injuries and health issues in athletes to improve their short- and long-term health and minimize downtime.

Due to the relatively young age of future professional athletes, prevention measures and medical consulting should be implemented in their early careers.

The purpose of this study is to conduct an exploratory analysis of hip flexibility and strength, dynamic body balance, functional movement and pelvic obliquity in elite junior riders to investigate their future equestrian-specific injury and health risks. This study will enable sports-specific injury prevention and improvements in future training concepts.

## Methods

### Study design and ethical considerations

This study was designed as a single-center cross-sectional study and approved by the institutional ethics committee at the University Hospital RWTH Aachen, Germany (EK 328 − 21). Written informed consent was obtained, and the rights of the participants have been protected. Inclusion in the study was voluntary, and medical care was not altered or influenced by study participation.

### Participants

Equestrian athletes from the 2021/2022 junior excellence cadre of Aachen Laurensberger Rennverein e.V. (ALRV), the organizer of the CHIO Aachen (World Equestrian Festival, Germany), were screened for participation in the study. The participants in the young elite talent program received training sessions in the form of riding lessons by the same professional riding instructors over a period of six months and, during this period, were evaluated and supported in terms of sports medicine by the Department of Orthopedics, Trauma and Reconstructive Surgery and the Institute for Physical Therapy of the University Hospital RWTH Aachen. The athletes trained for one week each month according to ALRV´s support program. At other times of the month, regular training continued at home. One day each month, the participants underwent a sports medicine examination at the University Hospital RWTH Aachen. The study design and analysis were not influenced by ALRV or the athletes.

### Inclusion and exclusion criteria

During the informed consent process, inclusion and exclusion criteria were reviewed before written informed consent was obtained.

Only adult participants (≥ 18 years) with no acute or active disease (including COVID-19) or pregnancy were screened for participation. The exclusion criteria included wearing a pacemaker, since doing so could interfere with the measurement instruments.

### Variables

The following variables were studied: demographic data (age, sex, height, weight), sports training data (discipline, training years in equestrian sports, training sessions and training hours per week), general injury or medical issues, fingertip-to-floor (FTF) test distances, pelvic obliquity, Y-balance test of the lower extremity (YBT-LQ) distances and scores, functional movement screen (FMS) scores, hip muscle strength of adductors and abductors, and Thomas test for M. iliopsoas and M. rectus femoris.

### Measurement

All measurements were performed at the Department of Orthopedics, Trauma and Reconstructive Surgery and the Institute of Physical Therapy by the same examiner. During the preceding interviews, medical histories were obtained with regard to previous traumatic sports injuries and overuse injuries that had a subjective influence on athletic performance. Chronic medical illnesses were a further point of inquiry. Each study day began with a warm-up procedure for 10 min, with participants alternating between exercise bike, recumbent bike, water rower, cross-trainer, and arm pedal trainer tasks every two minutes. All measurements for this study were performed following a standardized warm–up. Each examination day ended with 30 min of strength training on equipment and 30 min of instruction in stability and balance exercises for injury prevention [12].

On the first examination day, the biometric data and training behavior of the subjects were recorded, the finger-to-floor distance was measured (negative values were set as 0 cm and not measured more precisely), and pelvic obliquity was assessed and measured in centimeters (self-developed interview form available as a supplementary file).

Dynamic balance of the lower extremity was objectively measured with the YBT-LQ test using a Functional Movement Screen– Y Balance Test Kit (Perform Better Europe FTC Functional Training Company GmbH, Munich, Germany), as follow: one-leg stand; measurement of the maximum achieved distance of the meter on the ground pushed anteriorly, posteromedially and posterolaterally with the free leg; one trial round; and three main measurement rounds for both legs. Attempts that involved returning to the starting position from the maximum position with contact with the ground were scored as failed. For the composite score, the farthest distance for each direction on one side was added, and the sum was divided by three times the leg length. In addition, the difference between the distances achieved in a movement direction between the right and left sides was calculated. Consequently, the height of the subjects was not considered for the evaluation.

Functional flexibility was evaluated by the FMS using the Functional Movement Screen– Y Balance Test Kit according to the usual guidelines [13] with the following tasks: deep squat, hurdle step, inline lunge, shoulder mobility, active straight-leg raise, trunk stability push-up, and rotatory stability. For each exercise, no points were awarded for pain or a failed clearing-test, 1 point was awarded for incomplete execution, 2 points were awarded for a complete movement with errors or compensations, and 3 points were awarded for an ideal movement. The addition of all points resulted in the FMS score, which was considered with a frequent cut-off of 14 [14–16]. For double-sided tests, the lower value of both sides was used for the total score.

At the second examination appointment, the riders’ hip adductors and abductors were measured with the MusTec HD Force Evaluator (MusTec Muscle Dynamic Technology b.v., PC Almere, Netherlands). To avoid the problem of gravity compensation [17] and enable a direct comparison between adductors and abductors, a supine position was adopted. The force evaluator was placed 1 cm proximal to the knee condyles, and the subjects pressed the device laterally or medially against the examiner. From three measurements, the manufacturer’s operating program determined the maximum measured force.

On the sixth day of examination, shortening of the hip flexor muscles was examined using the modified Thomas Test for M. iliopsoas and M. rectus femoris for the right or left side according to the general specifications [18] with tightly controlled lumbopelvic movement to improve sensitivity and specificity [18].

### Statistical methods

Statistical analyses were performed between the following subgroups: dressage and show jumping; men and women; and (when available) the sides studied (right and left). Descriptive statistics were calculated for the subgroups and the total group. Continuous variables were described as mean and standard deviation (SD), while percentages were used to describe categorical variables.

All data except the results of the Thomas test were normally distributed. The study only considered main effects and no statistical interactions. To account confounding factors, the number of years of training in the equestrian sport was included as a covariate to identify an influence on the results (more variables were not possible due to incipient instability). Analysis of covariance (ANCOVA) was used to analyze continuous variables. For a side-by-side (right vs. left side) comparison of the continuous variables, a linear mixed model was used. Supplemental categorical variables were tested for significance using univariable logistic regression. In the case of a complete separation of data (results of the Thomas Test), the Firth method was used as an alternative to univariable logistic regression. The two-sided significance level *α* was set at 0.05. Adjustments to the significance level were not necessary because multiple testing was excluded. In the case of missing data, imputation or other statistical options were not applied. Statistical analysis was performed with the support of SPSS^®^ software Version 28 (IBM^®^ Inc., Armonk, NY) for Windows^®^.

## Results

### Participants

A total of 12 equestrian athletes were included in the analysis. Another participant in the training program could not be included due to being underage. Of these 12 participants, one male show jumping athlete complained of issues with his adductors, so adductor strength measurements were not performed for this participant. Two other male show jumping athletes were not able to attend the final day of testing due to COVID-19 pandemic restrictions, so no Thomas test could be performed for them.

### Descriptive data

**Fig. 1:**
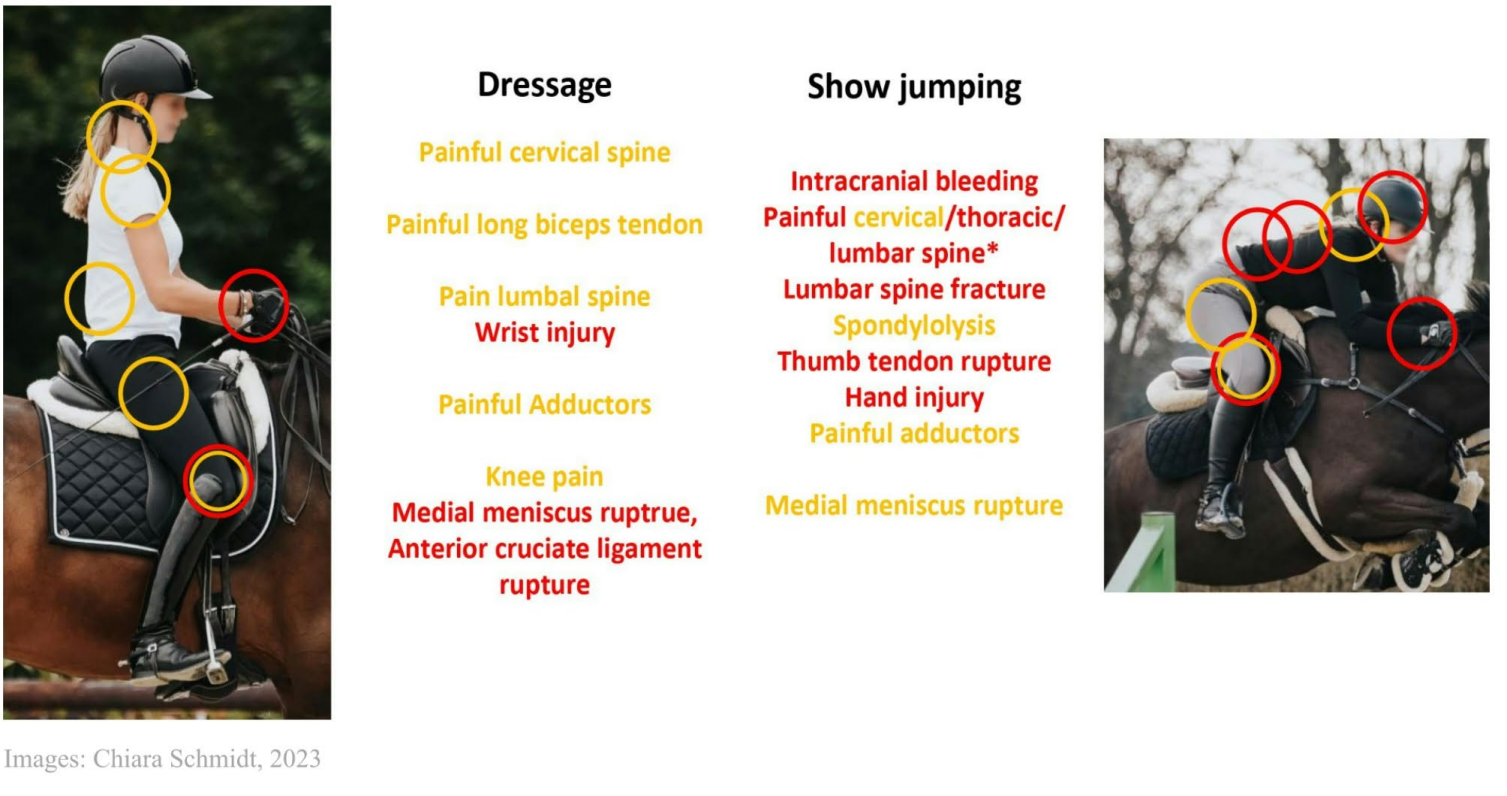
Compilation of orthopedic overuse injuries (yellow) and traumatic injuries (red)

Table 1 provides an overview of the descriptive data. Five of the 12 participants were dressage riders (41.7%), and seven were show jumpers (58.3%). The overall gender distribution was seven women (58.3%) and five men (41.7%), and it was balanced between the two disciplines, allowing for subdivision into the subgroups. The average BMI of all four subgroups was in the range for normal weight (BMI 18.5–24.9 kg/m^2^ [19]). Weight and height were significantly higher in male athletes than in female athletes. Among the riders, the mean number of years of training in equestrian sports (9.0 ± 3.7 years), the mean number of training sessions per week (9.9 ± 11.1 sessions), and the mean amount of training hours per week (23.1 ± 3.9 h) were determined. There were no significant differences between the subgroups. Ten athletes (83.3%) in the study group already showed general medical issues, and eight (66.8%) showed general past injuries, as summarized in Table 1. The localization and allocation of overuse and traumatic injuries are illustrated in Fig. 1. No significant influencing covariates could be identified.

**Table 1:** Biometrical and general data, history of medical issue or trauma

		Females	Males	*P* value sex	Show jumping	Dressage	*P* value discipline	Total	*P* value training years (confounding)
**Biometrical data**									
	Age, mean (SD), years	19.29 (0.8)	19.20 (1.3)	NA	19.43 (1.0)	19.00 (1.0)	NA	19.25 (1.0)	NA
	Female sex, No. (%)	NA	NA	NA	4 (57.1)	3 (60.0)	NA	7 (58.3)	NA
	Body height, mean (SD), cm	167.7 (3.9)	178.1 (7.7)	0.011*	170.3 (4.3)	174.5 (10.9)	0.116	170.0 (7.6)	0.231
	Weight, mean (SD), kg	58.6 (3.7)	67.2 (4.6)	0.006*	62.6 (5.6)	61.7 (7.0)	0.833	60.2 (5.9)	0.112
	BMI, mean (SD), kg/m²	20.8 (1.1)	21.3 (1.6)	0.627	21.5 (1.3)	20.3 (0.8)	0.172	21.0 (1.3)	0.621
**Training behavior**									
	Training years in equestrian sport, mean (SD), years	8.7 (3.6)	9.3 (4.1)	0.821	9.8 (4.6)	7.8 (1.5)	0.409	9.0 (3.7)	NA
	Training sessions per week, mean (SD), No.	6.5 (0.5)	14.6 (17.0)	0.236	12.1 (14.5)	6.8 (0.4)	0.766	9.9 (11.1)	0.73
	Training hours per week, mean (SD), hours	23.5 (4.7)	22.7 (3.0)	0.885	24.8 (3.9)	20.3 (1.5)	0.059	23.1 (3.9)	0.348
**History of medical issue or trauma**									
	Medical issue, No. (%), participants	5 (71.4)	5 (100.0)	0.999	6 (85.7)	4 (80.0)	0.767	10 (83.3)	0.824
	Trauma, No. (%), participants	4 (57.1)	4 (80.0)	0.165	6 (85.7)	2 (40.0)	0.424	8 (66.8)	0.377

Abbreviations: SD, Standard deviation; BMI, Body-Mass-Index

### Results of medical examinations/testing

The mean finger-to-floor distance was 11.7 ± 10.9 cm. Of the study population, 41.7% had pelvic obliquity, with an average of 1.1 ± 0.2 cm (Table 2) and all showing a lower pelvis on the right side than on the left side. Significant differences between the subgroups were not found.

**Table 2:** Fingertip-to-Floor (FTF) test, physical examination of the pelvis

	Females	Males	*P* value sex	Show jumping	Dressage	*P* value discipline	Total	*P* value training years (confounding)
FTF distance, mean (SD), cm	9.3 (9.7)	15.0 (12.8)	0.454	13.1 (12.3)	9.6 (9.5)	0.955	11.7 (10.9)	0.125
Pelvic obliquity, %	42.9	40.0	0.983	28.6	60.0	0.374	41.7	0.525
Deviation, mean (SD), cm	1.2 (0.3)	1.0 (0.0)	NA	1.0 (0.0)	1.2 (0.3)	NA	1.1 (0.2)	NA

Abbreviations: SD, standard deviation; FTF, Fingertip-to-Floor

For the YBT-LQ, mean composite scores between 86.4% and 91.0% were obtained for the subgroups (Table 3). The study group showed significant deviations (*p* =.031) of the mean values of the differences between the anteromedial distance for the right and left legs depending on the years of training in equestrian sports, and they were therefore positive for confounding. The average number of differences between right and left in one direction of movement varied between 0.1 and 5.4 cm. Strong differences in one direction of movement were found anteriorly in women (-3.7 ± 3.4 cm) and posteromedially in men (-4.0 ± 17.1 cm) and show jumpers (-5.4 ± 16.3 cm). All 12 subjects had a composite score below 94.0% on at least one side, while six subjects (50.0%) had a score below 94.0% on both sides.

**Table 3:** Lower quarter Y balance test

		Females	Males	*P* value sex	Show jumping	Dressage	*P* value discipline	Total	*P* value side influence	*P* value training years (confounding)
	Composite Score, mean (SD), %	91.0 (6.2)	86.4 (9.5)	0.171	89.2 (9.2)	90.1 (6.3)	0.858	89.6 (8.0)	0.614	0.957
**Difference between right and left**										
	composite score, mean (SD), %	-1.6 (8.4)	-1.3 (12.5)	NA	-4.4 (11.0)	2.6 (6.8)	NA	-1.5 (9.8)	NA	NA
	anterior distance, mean (SD), cm	-3.7 (3.4)	1.7 (9.8)	0.186	-2.4 (7.5)	-0.1 (6.7)	0.780	-1.5 (7.0)	NA	0.302
	posteromedial distance, mean (SD), cm	-0.5 (11.2)	-4.0 (17.1)	0.762	-5.4 (16.3)	2.9 (6.5)	0.610	-2.0 (13.3)	NA	0.031*
	posterolateral distance, mean (SD), cm	-0.8 (9.5)	-3.1 (9.1)	0.762	-5.1 (9.8)	2.9 (5.7)	0.281	-1.8 (9.0)	NA	0.055

Abbreviations: SD, standard deviation

The results of the FMS scores (Table 4) show that the mean values of the individual subgroups were above the pathological cut-off of 14 out of 21 points [14–16]. When evaluating the individual scores, there were two riders who scored less than 14 points. Only two subjects showed values of 1 (incomplete execution) in individual exercises; of these, a female jumper had a score of 1 for the active straight leg raise on both sides and a male jumper scored only 1 point for this exercise and also for the deep knee bend. There were no significant differences between the subgroups. The task trunk stability push-up proved positive for confounding with the years of training in equestrian sports (*p* =.044).

**Table 4:** Functional movement screen (FMS)

	Females	Males	*P* value sex	Show jumping	Dressage	*P* value discipline	Total	*P* value side influence	*P* value training years (confounding)
Deep Squat, mean (SD), points	2.3 (0.5)	2.0 (0.7)	0.491	2.1 (0.7)	2.2 (0.4)	0.787	2.2 (0.6)	NA	0.170
Hurdle Step, mean (SD), points	2.1 (0.4)	2.2 (0.4)	0.761	2.1 (0.3)	2.3 (0.5)	0.221	2.2 (0.4)	1.000	0.969
In-line Lunge, mean (SD), points	2.5 (0.5)	2.6 (0.5)	0.583	2.5 (0.5)	2.6 (0.5)	0.865	2.5 (0.5)	0.275	0.304
Shoulder Mobility, mean (SD), points	2.7 (0.5)	2.9 (0.3)	0.321	2.7 (0.5)	2.9 (0.3)	0.205	2.8 (0.4)	0.586	0.336
Active Straight-Leg Raise, mean (SD), points	2.1 (0.7)	2.0 (0.7)	0.800	1.9 (0.7)	2.4 (0.5)	0.297	2.1 (0.7)	1.000	0.263
Trunk Stability Push-up, mean (SD), points	2.3 (0.5)	2.4 (0.5)	0.556	2.4 (0.5)	2.2 (0.4)	0.165	2.3 (0.5)	NA	0.044*
Rotatory Stability, mean (SD), points	2.0 (0.0)	2.0 (0.0)	NA	2.0 (0.0)	2.0 (0.0)	NA	2.0 (0.0)	NA	NA
FMS-Score, mean (SD), points	15.1 ± 2.3	15.2 ± 1.5	0.818	14.4 ± 2.1	16.2 ± 1.3	0.259	15.2 ± 1.9	NA	0.126

Abbreviations: SD, standard deviation

For maximum hip adductor and abductor strength, there were significantly higher values in men than in women (adductors: *p* =.030, abductors: *p* =.010; comparative values in Table 5). In addition, adductor strength was significantly higher on the right side than on the left side (right: 30.41 ± 12.47 kg, left: 28.35 ± 12.02 kg; *p* =.046). The quotient of adductors to abductors amounted to an average of 1.14 ± 0.34 and was (like the strength of the adductors) significantly higher on the right side than on the left side (right: 1.22 ± 0.33, left: 1.06 ± 0.33; *p* =.028). 

**Table 5:** Hip muscle strength, stability and flexibility

	Females	Males	*P* value sex	Show jumping	Dressage	*P* value discipline	Total	*P* value side influence	*P* value training years (confounding)
Adductor muscle strength, mean (SD), kg	26.33 (12.30)	34.71 (9.97)	0.030*	29.13 (13.69)	29.67 (10.32)	0.890	29.38 (12.00)	0.046*	0.450
Abductor muscle strength, mean (SD), kg	22.91 (4.67)	29.69 (4.50)	0.010*	24.71 (6.01)	27.16 (5.04)	0.590	25.73 (5.65)	0.338	0.624
Adductor/abductor strength ratio, mean (SD)	1.12 (0.36)	1.16 (0.32)	0.719	1.19 (0.40)	1.08 (0.25)	0.873	1.14 (0.34)	0.028*	0.208
Thomas test for M. iliopsoas, No. (%)	4 (57.1)	1 (33.3)	0.981	5 (100.0)	0 (0.0)	0.014*	5 (50.0)	NA	NA
Thomas test for M. rectus femoris, No. (%)	4 (57.1)	1 (33.3)	0.981	5 (100.0)	0 (0.0)	0.014*	5 (50.0)	NA	NA

Abbreviations: SD, standard deviation

The Thomas test showed a complete separation of data between the dressage and show jumping riders. All show jumpers (100.0%) showed abnormalities for the iliopsoas and rectus femoris muscles (*p* =.014) compared to the dressage riders (0.0% abnormalities). No significant sex differences were found for the Thomas test (Table 5).

## Discussion

The study objective, the analysis of hip flexibility and strength, dynamic body balance, functional movement and pelvic obliquity, was successfully achieved.

The participants showed an age range of 18–21 years, due to the selected study populations of young rider. However, despite their young age, 66.7% already had a general history of injury, and 83.3% had a history of health issues. To prevent such injury and health issues, interventions need to be initiated at an early stage, so the young age of the test group seemed to be a good analytical opportunity for future therapeutic approaches.

High athletic specialization has been identified as a risk factor, with there being twice the risk of overuse injuries in highly specialized compared to low–specialized athletes [7, 8].

Based on the high number of years of training (9.0 years) and of training sessions per week (9.9 sessions) in relation to the average age (19.25 ± 1.0 years), a high level of athletic specialization could be deduced among our study population.

The determination of finger-to-floor distance has been characterized by an intra- and interobserver reliability of 0.99 for the measurement of hamstring flexibility and lumbopelvic movement [20]. Hamstring tightness has been correlated with lower back pain [21, 22] and can limit the lumbopelvic range of movement [22]. Guo et al. determined an ideal cut-off of the FTF distance of 2.6 to 8.8 cm [23]. Therefore, the mean values of the FTF test (11.7 ± 10.9 cm) did indicate abnormalities in our study population. Three participants had a finger-to-floor distance of above 20.0 cm. These three had no characteristics such as discipline or sex. Interpretatively, a conspicuous result with regard to hamstring tightness and the associated risk of lower back pain could be concluded for the study group [21, 22]. Other risk factors for low back pain that have already been identified for equestrian sports, such as trunk stability [24] and a limited range of movement of knee flexion and hip adduction [25], are crucial factors for the prevention and, therefore, should be included in a future assessment for more detailed evaluation of the low back pain risk. Larger study populations are needed to investigate the covariates and characteristics of hamstring tightness and to assess the potential need for a training focus for prevention.

Mendiguchia et al. [26] observed significant performance differences in sprinters with respect to their pelvic position. After targeted pelvic training to improve pelvic obliquity, their intervention group showed significantly faster sprint times. At 10.0%, with an average of 1.1 ± 0.2 cm, the proportion with pelvic obliquity observed in our study, should not be interpreted as conspicuous from a clinical perspective and did not seem to be of major importance to the young riders.

The YBT-LQ has been validated as a workable diagnostic tool for lower–extremity injury prevention [27] with good interrater test–retest reliability [28, 29]. Plisky et al. [30, 31] observed a 2.5-fold increased risk of lower extremity injury (no differentiation between traumatic and overuse injuries) in high school basketball players for deviations of 4 cm and more in one direction of movement and also demonstrated a 6.5-fold increased risk of injury in females with a composite score below 94.0%. Among the riders, a side difference of more than 4.0 cm was observed in men posteromedially and in show jumpers both posteromedially and posterolaterally. Furthermore, all subgroups had a composite score below 94.0%. Therefore, it can be assumed that all subgroups may have a risk factor for sport injuries. These observed asymmetries and low composite YBT-LQ scores in this sample suggest there are areas for intervention in equestrian athletes.

For the FMS, sex and discipline were not shown to be influencing factors, with 16.0% (one male and one female show jumper) having a score below 14. For this cut–off, Chang et al. [16] determined a 2.04-fold increased risk of sports injury in a sport-independent study group. Even if the explicit significance for equestrian riders has not yet been proven, a tendency, especially among show jumpers, becomes visible. Among the equestrian athletes observed, there were clinically relevant functional abnormalities that may pose an increased risk of injury.

Hip muscle strength was influenced by sex, with higher values in men. The disbalance of load on the adductors and abductors mentioned in the results can also be found in other sports. Tyler et al. [32] observed an opposite phenomenon (abductors loaded more than adductors) in ice hockey. There, athletes with an adductor–abductor strength ratio of less than 0.8 were found to have a 17-fold increased risk of injury for the following season. Assuming comparable deviations of 20.0% in the direction of stronger adductors, the observed averages (1.08–1.19) in the riders may also indicate a risk of injury. The significantly stronger right adductors observed possibly increased this vulnerability due to the imbalanced ratio among equestrian athletes.

From the clinical experience of orthopedic surgeons and physiotherapists working with the CHIO Aachen for a long period of time, the reason for the complete separation with the modified Thomas test between dressage (all negative) and jumping (all positive) was not clear. Sex had no influencing effect. Possible causes could be the different leg–hip position due to the different saddles and longer stirrups or different movement patterns between these two disciplines (leg position is visible in Fig. 1) resulting in a flatter lumbar alignment for show jumpers [33]. In particular, the reason for existing hip flexor contractures in show jumpers and hamstring contractures in the overall group should be further investigated. This investigation should be combined with an analysis of the spinal morphotype, to provide a complete overview of the complex relationship concerning a risk factor assessment of overuse and traumatic injuries in equestrian sports.

### Limitations and generalizability

Most of the tests used in this study have only been evaluated for other sports or have been tested in a non-sport-specific way, so the external validity of the tests specifically for equestrian sports should be checked in the future. Moreover, most studies examining the utilized tests did not assess the potential impact of prior injuries (either traumatic or overuse-related) on the prospective risk assessment. Consequently, future scientific investigations should address this gap in knowledge and differentiate between prior injuries and future risk factors.

The results of the study are not applicable to all equestrian athletes. A review of their generalizability to all performance levels and all age groups should also be conducted, as this study focused on young athletes at a more experienced riding level. Due to the homogeneous sex distribution, the results are transferable to men and women.

The small sample size of this study severly limits the generalizability of the results. Especially an analysis looking for significant covariates can only be interpreted with major limitations. All results and interpretations should be reproduced and expanded in future specialised studies with a larger sample size.

## Conclusion

Despite their young age, the junior riders showed a high degree of athletic specialization, a high proportion of previous injuries and medical issues, signs of hamstring tightness, risk constellations on the YBT-LQ, hip strength disbalance and for all show jumpers signs of hip flexion contraction.

Consequently, the dressage and show jumping riders could have high potential for injury. The problems identified should be explicitly investigated in further equestrian sports studies for potential future training improvements and injury risk reduction.

## Electronic supplementary material

Below is the link to the electronic supplementary material.

Survey and examination

## Data Availability

The datasets used and/or analysed during the current study are available from the corresponding author on reasonable request. The self-developed interview form is available as a supplementary file.

## Abbreviations

ALRV: Aachen laurensberger rennverein e.V
ANCOVA: Analysis of covariance
BMI: Body-mass-index
FMS: Functional movement screening
FTF: Fingertip-to-floor
SD: Standard deviation
YBT-LQ: Lower quarter Y-balance testing
