# Supplementary material for: Sports injury risk assessment based on a training and functional movement analysis of young elite equestrian athletes– an exploratory cross-sectional study
Source: BMC Sports Sci Med Rehabil. 2025 Apr 14;17:83. doi: 10.1186/s13102-025-01138-x (PMC11995532; doi:10.1186/s13102-025-01138-x)
Supplement: Supplementary file 1 — Survey and examination [file 13102_2025_1138_MOESM1_ESM.pdf]

Department of Orthopedics, Trauma and Reconstructive Surgery
Medical Faculty, RWTH Aachen University,
Pauwels Strasse 30, 52074 Aachen, Germany

Survey and examination: CHIO Young Elite Equestrian Athletes

Date: ____________________
Examiner: ____________________
Probands ID: ____________________

Biometric data
Age: ____________________
Sex: ____________________
Height: ____________________
Weight: ____________________

Equestrian data
Discipline: ____________________
Training years in equestrian sports: ____________________
Training sessions per week: ____________________
Training hours per week: ____________________

Medical history
General injuries: ____________________
Medical issues: ____________________

Physical Examination
Pelvic obliquity: ____________________
Finger-to-floor distance: ____________________
